# HIV latency reversing agents act through Tat post translational modifications

**DOI:** 10.1186/s12977-018-0421-6

**Published:** 2018-05-11

**Authors:** Georges Khoury, Talia M. Mota, Shuang Li, Carolin Tumpach, Michelle Y. Lee, Jonathan Jacobson, Leigh Harty, Jenny L. Anderson, Sharon R. Lewin, Damian F. J. Purcell

**Affiliations:** 1 Department of Microbiology and Immunology, The Peter Doherty Institute for Infection and Immunity, University of Melbourne, Melbourne, Australia; 2 The Peter Doherty Institute for Infection and Immunity, Royal Melbourne Hospital, University of Melbourne, Melbourne, Australia; 3 School of Life Sciences, Peking University, Beijing, China; 4 Department of Infectious Diseases, Alfred Health and Monash University, Melbourne, Australia

**Keywords:** HIV latency, LRA, Tat, Post-translational modification, Splicing

## Abstract

**Background:**

Different classes of latency reversing agents (LRAs) are being evaluated to measure their effects in reactivating HIV replication from latently infected cells. A limited number of studies have demonstrated additive effects of LRAs with the viral protein Tat in initiating transcription, but less is known about how LRAs interact with Tat, particularly through basic residues that may be post-translationally modified to alter the behaviour of Tat for processive transcription and co-transcriptional RNA processing.

**Results:**

Here we show that various lysine and arginine mutations reduce the capacity of Tat to induce both transcription and mRNA splicing. The lysine 28 and lysine 50 residues of Tat, or the acetylation and methylation modifications of these basic amino acids, were essential for Tat transcriptional control, and also for the proviral expression effects elicited by histone deacetylase inhibitors (HDACi) or the bromodomain inhibitor JQ1. We also found that JQ1 was the only LRA tested that could induce HIV mRNA splicing in the absence of Tat, or rescue splicing for Tat lysine mutants in a BRD4-dependent manner.

**Conclusions:**

Our data provide evidence that Tat activities in both co-transcriptional RNA processing together with transcriptional initiation and processivity are crucial during reactivation of latent HIV infection. The HDACi and JQ1 LRAs act with Tat to increase transcription, but JQ1 also enables post-transcriptional mRNA splicing. Tat residues K28 and K50, or their modifications through acetylation or methylation, are critical for LRAs that function in conjunction with Tat.

**Electronic supplementary material:**

The online version of this article (10.1186/s12977-018-0421-6) contains supplementary material, which is available to authorized users.

## Background

The major barrier to a cure for HIV is long lived latently infected memory CD4+ T-cells that persist on antiretroviral therapy (ART) [1]. One strategy being investigated to eliminate latency is to activate virus production from latency in the presence of ART so that no further rounds of infection occur, and it was speculated that the cell would then die either through immune mediated clearance or virus induced cytolysis [1]. Epigenetic modifiers including histone deacetylase inhibitors (HDACi) have been used to reverse latency in vitro and in vivo [2–7]. Although clinical trials of these agents in HIV-infected individuals on ART demonstrated modest increases in cell-associated unspliced HIV mRNA (US RNA), indicative of the initiation of viral transcription, when used alone, these studies failed to show a reduction in the frequency of latently infected cells as measured by HIV DNA [2–7]. Understanding how different classes of latency reversing agents (LRAs) affect distinct aspects of virus production post integration is needed to define the optimal compounds to efficiently reverse latency.

Tat is a critical viral protein required to transactivate viral transcriptional elongation and splicing [8–13]. In active HIV replication, Tat undergoes various post-translational modifications including acetylation and methylation. Depending on which residue is modified and the type of modification it carries, the behaviour of Tat changes to regulate its activity [14–27]. These multiple modifications provide an interconnected regulatory network that enables Tat to control viral transcription, elongation, and splicing throughout viral replication [14–27]. These modifications differ depending on the cellular environment, specifically the activation state of the cell [16], and thus should differ during active replication in activated cells versus latent infection in resting cells. Moreover, these modifications may differ under the influence of LRAs as a limited number of studies have demonstrated additive effects of LRAs with the viral protein Tat in initiating transcription [28]. However, less is known about how LRAs interact with Tat, particularly through basic residues that may be post-translationally modified to alter the behaviour of Tat.

In this study, we generated a series of Tat mutants to determine their effects on viral transcription and/or splicing. We investigated the effects of Tat mutants on the activity of a panel of LRAs and found that post translational modifications of different lysine residues of Tat are important for its activity with different LRAs, with differing abilities to actively initiate transcription and/or splicing.

## Results

### A novel in vitro model using fluorescent reporter proteins to test the impact of interventions on HIV transcription and splicing

A potent LRA is required for efficient reactivation and clearance of latent proviruses. To investigate the ability of LRAs to induce HIV-1 transcription and splicing, we determined the effects of LRAs alone or in combination with full-length wild-type (WT) Tat101 given Tat’s important role in splicing and transcription [8].

**Fig. 1:**
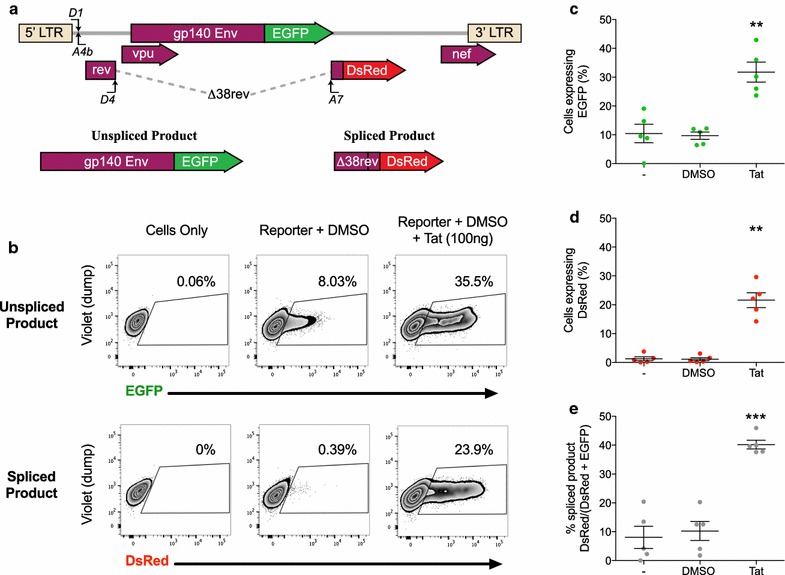
Model used to determine the effects of LRAs on LTR-driven transcription and splicing in the presence and absence of Tat. **a** Schematic of the in vitro model used in this study to determine the effects of LRAs on LTR-driven transcription and splicing. HEK293T cells were co-transfected with the LTR reporter construct together with a plasmid expressing Rev, with or without a plasmid expressing Tat for 48 h, followed by analysis using flow cytometry. The LTR construct expresses either unspliced protein fused to enhanced green fluorescent protein (EGFP) or spliced ∆38rev protein fused with DsRed. **b** Gating strategy of a representative sample used to identify the percentage of cells expressing EGFP or DsRed. **c**–**e** The combined results of 5 independent experiments showing the percentage of cells expressing EGFP (**c**), DsRed (**d**) or the percentage of spliced products (DsRed/DsRed+ EGFP) (**e**). The mean ± SEM of the 5 separate experiments, each run in triplicate, is shown. Comparisons were made to cells only (−) using a paired *T* test. Only statistically significant comparisons are shown **p < 0.01; ***p < 0.001

To this end, we developed an in vitro model that can distinguish between unspliced and spliced viral products by the expression of EGFP and DsRed fluorescent proteins respectively (Fig. 1a). Briefly, a subgenomic reporter construct pLTR.gp140/EGFP.Rev∆38/DsRed, which derives from the authentic *env2* mRNA [29], was constructed to allow the detection of LTR-driven ‘unspliced’ or ‘spliced’ products by flow cytometry. This construct expresses HIV-1 Env, Rev and small amounts of Vpu proteins [30, 31]. In this system if the mRNA remains unspliced, it would express Env (gp140) fused to EGFP (Fig. 1a). If splicing occurs across splice donor 4 and splice acceptor 7 (D4A7), the spliced mRNA encodes a non-functional Rev protein truncated at amino acid 38 fused to DsRed fluorescent protein (Δ38Rev-DsRed).

To test that this model measures unspliced and spliced viral products, HEK293T cells were co-transfected with the reporter and a plasmid containing rev (pRev) to facilitate the nuclear export of Rev response element (RRE)-containing unspliced mRNA, in the presence or absence of pTat101. Flow cytometry was used to measure each fluorescent colour using the gating strategy shown for a representative sample (Fig. 1b) out of five independent experiments, each performed in triplicate (Fig. 1c–e). For cells transfected with the reporter and pRev without Tat, a mean of 8.03 and 0.39% of cells expressed EGFP and DsRed respectively, indicating that there was basal transcription from the LTR but only a low efficiency of splicing at D4A7 without Tat (Fig. 1c–e, minus sample). The addition of WT Tat101 (100 ng pTat) enabled a significant increase in transcription (31.7% EGFP+; p = 0.0076) (Fig. 1c) and splicing (21.6% DsRed+; n = 5, Paired *T* test, p = 0.0015) (Fig. 1d). To control for Tat-induced increases in transcription and for transfection efficiency, we also measured the percentage of spliced product versus total product (spliced/(spliced + unspliced) × 100). A significant increase in spliced product was observed in the presence of WT Tat compared to cells only and DMSO (p = 0.0004; n = 5; Paired *T* test) (Fig. 1e).

### Latency reversing agents change the behaviour of Tat during reactivation from the LTR

Using the same reporter system, we investigated the effects of a panel of LRAs on LTR-driven transcription and splicing in the absence and presence of WT Tat. Transfected cells were treated with the two HDACi vorinostat (VOR) and panobinostat (PAN), the bromodomain inhibitor JQ1, the methyltransferase inhibitor chaetocin (CTN), the anti-alcoholic disulfiram (DIS) and the T cell activation stimuli phorbol myristate acetate/phytohaemagglutinin (PMA/PHA), with concentrations at the maximum tolerated doses to preserve cell viability (Additional file 1: Fig. S1). Cells were then harvested at 48 h post-transfection for flow cytometry analysis.

**Fig. 2:**
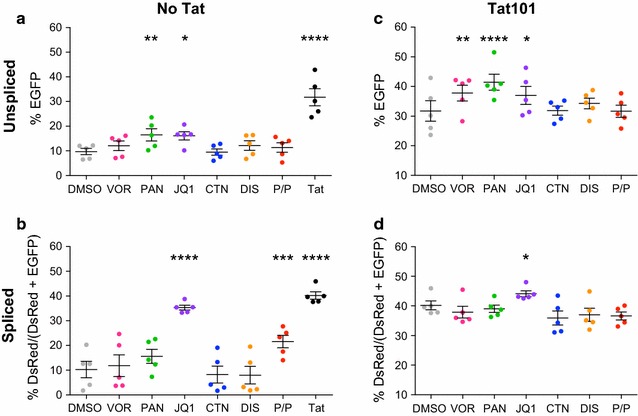
JQ1 but not HDACi can increase splicing in the absence and presence of Tat. HEK293T cells were co-transfected with the pLTR.gp140/EGFP.Rev∆38/DsRed splicing reporter together with a plasmid expressing Rev, in the absence (**a**, **b**) or presence (**c**, **d**) of 100 ng of pTat101 (AD8)-Flag expression plasmid and then treated with a panel of LRAs or DMSO diluent control (n = 5). EGFP (unspliced) and DsRed (spliced) expression were measured using flow cytometry. Comparisons of each condition to DMSO were made using 2-way ANOVA test. Only statistically significant comparisons are shown *p < 0.05; **p < 0.01; ***p < 0.001; ****p < 0.0001. The black lines represent the mean ± SEM. DMSO (1:5000), VOR = vorinostat (0.5 μM), PAN = panobinostat (30 nM), JQ1 (+) (1 μM), CTN = chaetocin (30 nM), DIS = disulfiram (500 nM), or PMA/PHA = phorbol myristate acetate/phytohaemagglutinin (10 nM PMA, 10 μg/mL PHA)

Without Tat, both PAN and JQ1 were the only LRAs that significantly increased the expression of EGFP (1.68 and 1.69 FC over DMSO respectively; n = 5, 2-way ANOVA across LRAs, p < 0.05) indicative of unspliced transcripts (Fig. 2a). However, this increase was modest compared to Tat when added *in trans* (3.27 FC over DMSO, 2-way ANOVA, p = 0.0001) (Fig. 2a). Moreover, in the absence of Tat, JQ1 was the only LRA that increased the proportion of spliced product to a similar level as WT Tat (3.44 and 3.92 FC over DMSO respectively; n = 5, 2-way ANOVA, p = 0.0001) (Fig. 2b).

In the presence of Tat101, while JQ1 and both HDACi significantly increased the levels of EGFP (1.2 and 1.3 FC over DMSO respectively, p < 0.05) (Fig. 2c), only JQ1 significantly increased the proportion of spliced product when compared to DMSO (Fig. 2d; n = 5, 2-way ANOVA across all LRAs). Similar results were obtained using Tat86 from the HIV NL4-3 strain (data not shown). Taken together, these data demonstrate that different classes of LRAs have differential effects on HIV transcription and splicing. In addition, while Tat alone is the most potent activator of LTR-driven transcription, Tat can also have an additive effect with HDACi and JQ1 on transcription, and with JQ1 alone on splicing.

### Accumulation of HIV spliced RNA following JQ1 treatment

**Fig. 3:**
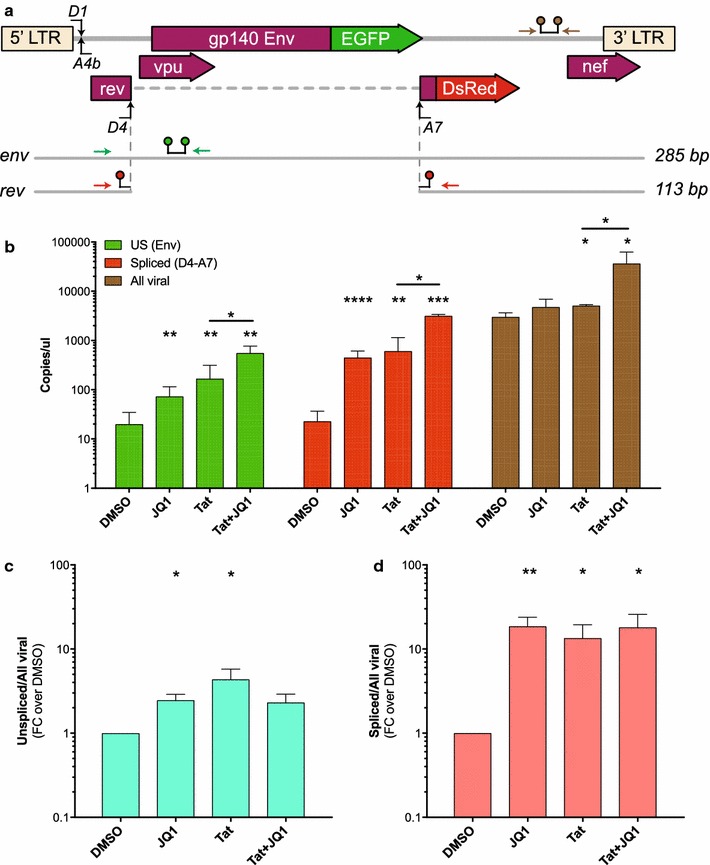
JQ1 induces HIV spliced RNA accumulation in the absence and presence of Tat. **a** Schematic of unspliced and spliced mRNAs produced following HEK293T cell transfection with the pLTR.gp140/EGFP.Rev∆38/DsRed splicing reporter. The different sets of primers-probe used for HIV RNA quantification are displayed as coloured arrows for primers, and linked coloured spots for the probe. The *env* set detects unspliced mRNA (green), *rev* set detects spliced mRNA (red) and the viral set detects all viral mRNAs including the co-transfected pRev^NL4.3^ mRNA (brown). **b**–**d** HEK293T cells transfected in the absence or presence of 100 ng of pTat101 (AD8)-Flag expression plasmid were treated for 24 h with JQ1 (1 μM) or DMSO diluent control (n = 4). Cells were then harvested and EGFP (unspliced) and DsRed (spliced) protein expression measured using flow cytometry (Fig. S2), while HIV unspliced (US), spliced (D4-A7) and all viral RNA expression levels (copies/μl) were quantified by droplet digital PCR (ddPCR) (**b**–**d**). The ratio of US (**c**) and spliced (**d**) over all viral RNAs is displayed as fold-change (FC) over DMSO. Comparisons of each condition to DMSO were made using a paired *T* test. Only statistically significant comparisons are shown *p < 0.05; **p < 0.01; ***p < 0.001; ****p < 0.0001. The black lines represent the mean ± SEM

To validate the effect of JQ1 on HIV-1 transcription and splicing, we performed similar experiments but also evaluated the changes in viral RNA levels following treatment with JQ1 in the presence or absence of Tat (Fig. 3, Additional file 2: Fig. S2). Transfected cells were harvested at 24 h to capture the peak of RNA expression. Most cells were used for RNA quantification by droplet digital PCR (ddPCR, Fig. 3), while a small portion of cells were also analysed by flow cytometry (Additional file 2: Fig. S2). For ddPCR, a specific probe spanning D4-A7 splice sites was used to quantify the spliced RNAs (Fig. 3a, *rev* primer–probe). Unspliced (US) transcripts were detected by a primer–probe set specific to gp140 Env ORF (Fig. 3a, *env* primer–probe set). Additionally, we quantified all viral RNAs initiated from the HIV long terminal repeat (LTR) by targeting a common region of viral transcripts (Fig. 3a, virus primer–probe).

Both JQ1 alone and Tat alone increased the levels of US (3.16 and 6.65 FC over DMSO) as well as spliced RNA (19.75 and 19.33 FC over DMSO), while no significant change was detected in the level of total viral RNA following JQ1 treatment (Fig. 3b). As we observed previously in Fig. 2, the combination of Tat and JQ1 increased the levels of US and spliced RNAs (Fig. 3b) confirming the ability of JQ1 and Tat to turn on HIV transcription and splicing. Moreover, the RNA analysis revealed consistent changes in RNA transcription and splicing that was mirrored by the EGFP and DsRed protein expression in this model following treatment with JQ1: a 2.5-fold increase in both US/all viral RNA and %EGFP+, and 18.5-fold increase in spliced/all viral RNA and %spliced product (compare Fig. 3c, d and Additional file 2: Fig. S2A, S2C). This indicates that EGFP and DsRed expressing cells well reflect the ability of JQ1 to induce HIV transcription and splicing at D4A7. These results also confirm the ability to use the splicing reporter pLTR.gp140/EGFP.Rev∆38/DsRed in high throughput assays where we can accurately measure by flow cytometry the effect of LRAs on HIV transcription and splicing.

**Fig. 4:**
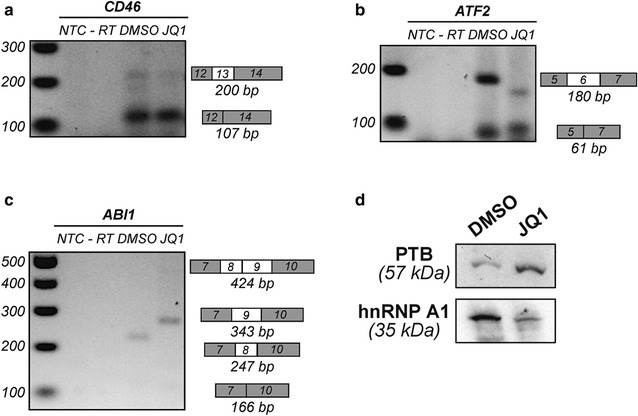
JQ1 affects alternative splicing by modulating hnRNP protein levels. Alternative splicing pattern of *CD46* (**a**), *ATF2* (**b**) and *ABI1* (**c**) in HEK293T cells following 24 h of JQ1 (1 μM) or DMSO treatment. Inclusion and exclusion of exons are indicated on the right of the gel with white boxes, while grey boxes represent constitutively included exons. NTC = No Template Control, -RT corresponds to minus reverse transcriptase control. **d** Western Blot analysis of splicing factors following treatment with JQ1 or DMSO diluent control

To determine whether this was a global effect on cellular RNA transcription, we also looked at changes in *RPP30*, *IPO8* and *TBP* RNA levels following each treatment (Additional file 3: Fig. S3A) and saw no statistically significant changes in HIV RNA fold changes when normalized to all 3 references genes (Additional file 3: Fig. S3B). We next evaluated whether the effect of JQ1 was a general splicing effect or specific to HIV D4A7 sites by looking at the alternative splicing pattern of multiple human pre-mRNA including *CD46*, *ATF2* and ABI-interactor (*ABI1*) by RT-PCR using specific primers of adjacent exons. A reduction in *CD46* exon 13 (Fig. 4a) and *ATF2* exon 6 (Fig. 4b) inclusions was observed following treatment with JQ1. In addition, *ABI1* exon 8 exclusion and exon 9 inclusion were favoured in the presence of JQ1 (Fig. 4c) suggesting a global effect of JQ1 on alternative splicing. Finally to determine whether the increase in HIV D4-A7 splicing was due to changes in the availability of splicing factors, we also looked at changes in hnRNP protein levels following JQ1 treatment. An increase in PTB coupled with a decrease in hnRNP A1 levels was observed (Fig. 4d).

Overall, these data demonstrate that JQ1 treatment leads to a significant accumulation of HIV spliced RNA. This was consistent with an increase in the ability of JQ1 to drive both HIV-1 transcription but more profoundly splicing, potentially due to a decrease in splicing inhibitory factors such as hnRNP A1 and an increase in RNA binding proteins facilitating RNA nuclear export like PTB.

### Mutations in lysine and arginine residues in Tat101 reduce HIV transcription and splicing efficiency

**Fig. 5:**
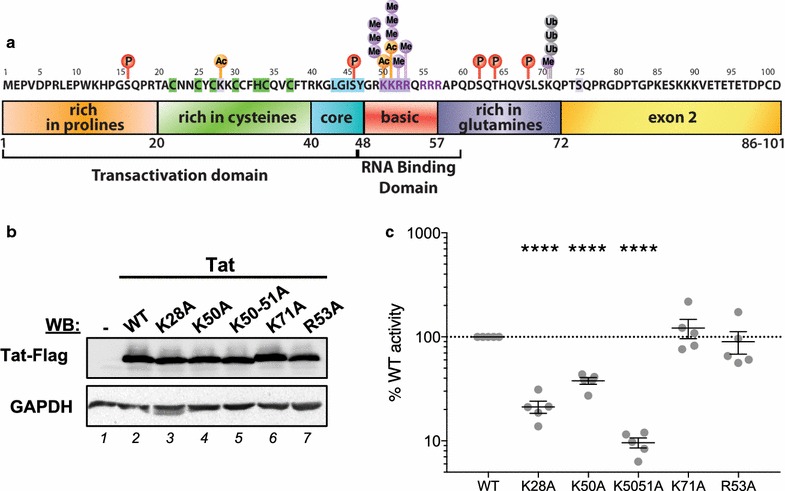
Mutations in Tat at lysine and arginine residues reduce the efficiency of HIV transcription. **a** Schematic diagram showing Tat protein sub-domains and residues that are post-translationally modified. Specific basic lysine (K) and arginine (R) residues were mutated to alanine (A) in pTat101 (AD8)-Flag using site-directed mutagenesis. **b** Western Blot analysis of wild-type (WT) or mutant Tat expression in HEK293T cells 48 h post-transfection using anti-Flag antibody. GAPDH was used as a loading control. **c** Luciferase expression of TZMbl cells 48 h following transfection of Tat mutants compared to WT Tat101, represented as the  % of WT activity. Only statistically significant comparisons are shown ****p < 0.0001 (Paired *T* test). The black lines represent the mean ± SEM (n = 5). For the letters in coloured circles: *P* phosphorylation, *Ac* acetylation, *Me* methylation, *Ub* ubiquitination

Transactivation of the HIV-1 LTR by Tat is a tightly controlled process that is heavily reliant on post-translational modifications (PTMs) of both Tat and P-TEFb [16]. These PTMs serve to fine-tune Tat function allowing its interaction with different cofactors at specific stages of transcription. Several Lysine (K) and Arginine (R) residues in the transactivation and RNA binding domains of Tat are modified (Fig. 5a). To measure the effect of PTMs on HIV transcription and splicing, we introduced a variety of mutations in the basic K or R residues of Tat, using site-directed mutagenesis to change these residues into Alanine (A). The Tat proteins included a C-terminal flag-tag, which allowed their detection by Western blot using an anti-Flag antibody (Fig. 5b). Each mutant or wild-type (WT) Tat was first transfected into HEK293T cells and Western blot confirmed similar expression levels of these mutants as WT Tat (Fig. 5b).

Next, we investigated the effects of these mutations on LTR-driven transcription alone (without splicing) by transfecting each mutant into TZMbl cells that contain a luciferase reporter under the control of HIV LTR. Cells were harvested 48 h later and luciferase expression was measured and represented as a percentage of expression relative to WT Tat (Fig. 5c). Mutations of K28, K50 and K50-51 to alanine significantly reduced the ability of Tat to transactivate transcription from the LTR, while no effect was observed following K71 and R53 mutations (n = 5; Paired *T* test).

**Fig. 6:**
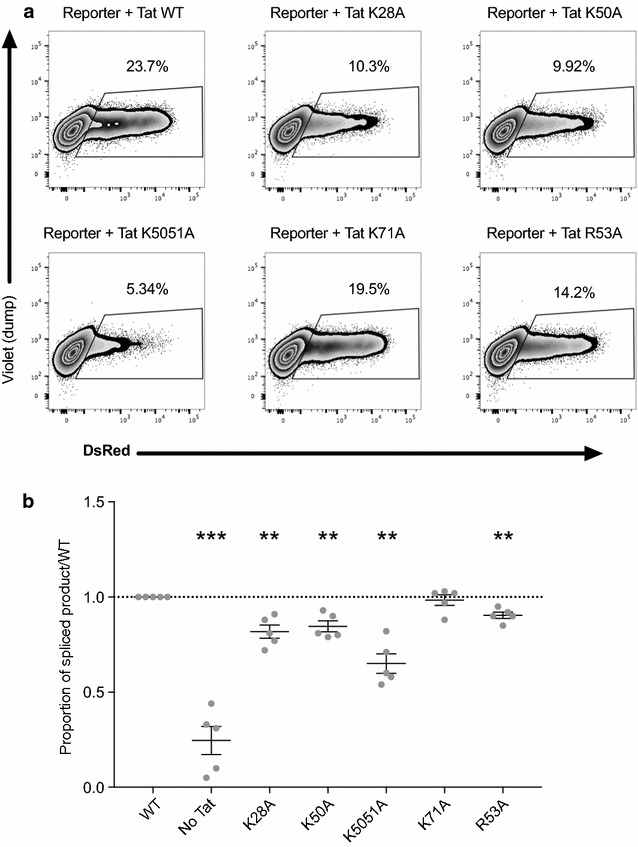
Specific mutations within Tat reduce HIV D4-A7 splicing. HEK293T cells were co-transfected with the pLTR.gp140/EGFP.Rev∆38/DsRed splicing reporter, 20 ng of pRev and 100 ng of pTat101 without (WT) or with specific mutations (K28A, K50A, K50/51A, K71A and R53A). Cells were harvested at 48 h and DsRed expression was quantified by flow cytometry. **a** A representative example of the gating strategy used to identify  % DsRed positive cells that represent spliced product. **b** The proportion of spliced product [DsRed/(DsRed + EGFP) × 100] relative to WT Tat is shown from n = 5 independent experiments, each conducted in triplicate. Comparisons of each condition to DMSO were made using a Paired *T* test. Only statistically significant comparisons are shown **p < 0.01; ***p < 0.001. The black lines represent the mean ± SEM

Similar results were obtained when Tat mutants were co-transfected with the splicing reporter construct to assess effects on splicing (Fig. 6). In this set of experiments, these same mutants also exhibited statistically significant reductions in the proportion of spliced product (Fig. 6; n = 5; Paired *T* test). These data demonstrate that mutations to specific lysine residues within Tat significantly inhibit the efficiency of Tat in driving transcription and splicing at D4A7.

### Lysine 28 and 50 mutants reduce LTR-driven transcription in the presence of LRAs

Next, to investigate whether Tat PTMs affect LTR-driven transcription with commonly used LRAs, we co-transfected each mutant into HEK293T with the splicing reporter described above and then treated the cells with the LRA panel or DMSO diluent control.

To assess the effects of Tat PTMs on transcription (EGFP+ cells), statistical comparisons of EGFP+ cells were made to DMSO+ Tat101 with or without the designated mutation (Additional file 4: Fig. S4). VOR, PAN and JQ1 had statistically significant activity with WT Tat101 (Additional file 4: Fig. S4A; n = 5; 2-way ANOVA test across all LRAs). However, VOR and JQ1 lost this significant activity with the K28A and K50A mutants compared to their respective DMSO control (Additional file 4: Fig. S4B and S4C). In contrast, Tat K50/51A retained its activity with HDACi and JQ1, and gained a modest but statistically significant enhancement of activity with PMA/PHA (Additional file 4: Fig. S4C). The pattern was different with the K71A and R53A mutants, where PAN and JQ1 retained their ability to induce transcription, but this ability was lost with VOR (Additional file 4: Fig. S4E-F). These data suggest that Tat K28 and K50, or the post translational modifications that these basic amino acids acquire, are essential for Tat action with VOR and JQ1 in driving transcription.

### JQ1 promotes HIV D4-A7 splicing with or without WT Tat101 and its various mutants in a splicing reporter system

**Fig. 7:**
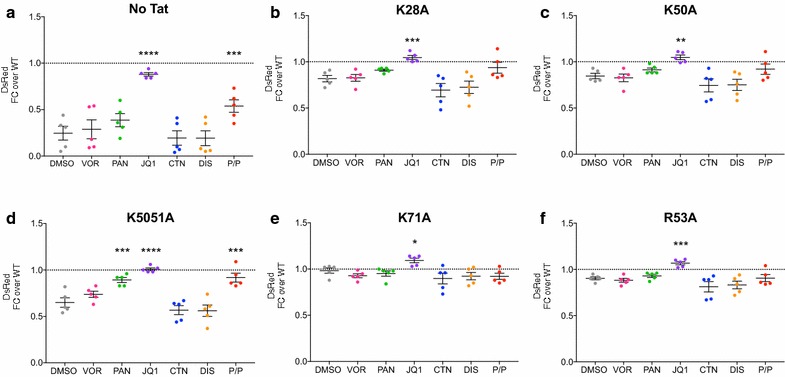
JQ1 consistently rescued HIV D4A7 splicing. HEK293T cells were transfected with the pLTR.gp140/EGFP.Rev∆38/DsRed splicing reporter without (**a**) or with 100 ng of pTat101 (AD8)-Flag that was either wild-type (shown in Fig. 2) or had the specific mutations: **b** K28A, **c** K50A, **d** K50/51A, **e** K71A or **f** R53A. Transfected cells were then treated with a panel of LRAs for 48 h, harvested and DsRed expression was quantified using flow cytometry. The fold change in the proportion of spliced product [DsRed/(DsRed + EGFP) × 100] in the no Tat or Tat mutants relative to WT Tat+ DMSO is represented. Comparisons of each condition to DMSO were made using the 2-way ANOVA test. Only statistically significant comparisons are shown *p < 0.05; **p < 0.01; ***p < 0.001; ****p < 0.0001. The black lines represent the mean ± SEM. DMSO (1:5000), VOR = vorinostat (0.5 μM), PAN = panobinostat (30 nM), JQ1 (+) (1 μM), CTN = chaetocin (30 nM), DIS = disulfiram (500 nM), or PMA/PHA = phorbol myristate acetate/phytohaemagglutinin (10 nM PMA, 10 μg/mL PHA)

**Fig. 8:**
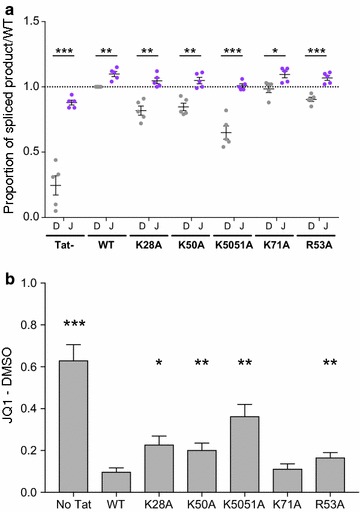
JQ1 can rescue the splicing activity lost by Tat mutants. HEK293T cells were transfected with the pLTR.gp140/EGFP.Rev∆38/DsRed splicing reporter and 100 ng of pTat101 (AD8) WT or with specific mutations (K28A, K50A, K50/51A, K71A and R53A). Cells were treated with DMSO (D, 1:5000) or JQ1 (J, 1 μM), harvested at 48 h and DsRed and EGFP expression quantified using flow cytometry. **a** Data were represented as the proportion of spliced product [DsRed/(DsRed + EGFP) × 100] relative to WT Tat+ DMSO. Comparisons between DMSO and JQ1 for either no Tat, WT Tat or each mutant Tat were made using multiple paired *T* tests. **b** The difference between JQ1 and DMSO in the proportion of spliced product [DsRed/(DsRed + EGFP) × 100] was calculated for each Tat mutant, no Tat or WT Tat, and then compared to the difference with WT Tat101. Only statistically significant comparisons are shown *p < 0.05; **p < 0.01; ***p < 0.001. The black lines represent the mean ± SEM (n = 5)

To assess the effects of the Tat mutants on HIV splicing (DsRed cells), the HEK293T cells transfected and treated with a panel of LRAs above were analysed by flow cytometry for the percentage of cells expressing DsRed. The fold change in the proportion of spliced product [spliced/(spliced + unspliced)/WT] of Tat mutants relative to WT Tat101+ DMSO is represented in Fig. 7. Although both PAN and JQ1 increased the percentage of cells expressing the unspliced EGFP product without or with WT Tat (Fig. 2), only JQ1 consistently increased the proportion of DsRed spliced product in the absence of Tat (Fig. 7a) and with each Tat mutant (Fig. 7b–f; n = 5; 2-way ANOVA test across all LRAs). We directly compared these changes to DMSO (Fig. 8a), and displayed the difference in activity of JQ1 within each mutant and found statistically significant differences compared to WT Tat101. Although in each mutant, JQ1 enabled a statistically significant increase in the proportion of spliced product compared to DMSO that was always greater than WT activity (Fig. 8a; n = 5; Paired *T* test), when compared to WT Tat101 this difference was significantly higher for all mutants except K71A (Fig. 8b; n = 5; Paired *T* test). Taken together, these data show that the presence of WT Tat or JQ1 alone can both enable HIV RNA splicing to a similar degree, and when a specific Tat mutation reduces splicing efficiency (Fig. 6b), only JQ1 could rescue splicing (Figs. 7, 8). Thus, JQ1 may act directly or indirectly with Tat, and independently of the basic K or R residues of Tat, to enable HIV RNA splicing.

### BRD4 mediates JQ1 effect on HIV D4-A7 RNA splicing

**Fig. 9:**
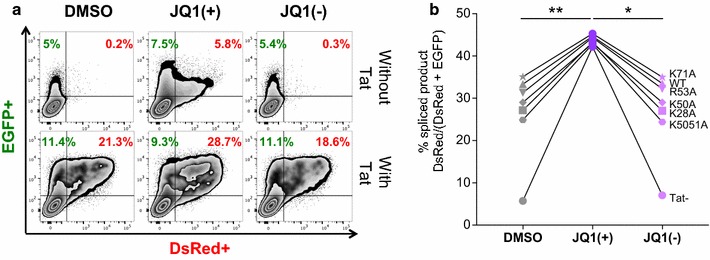
BRD4 mediates HIV D4-A7 RNA splicing. HEK293T cells were co-transfected with the pLTR.gp140/EGFP.Rev∆38/DsRed splicing reporter, 20 ng of pRev and 100 ng of pTat101 (AD8) without (wild-type, WT) or with specific mutations (K28A, K50A, K50/51A, K71A and R53A) then treated with DMSO, 1 μM JQ1 (+) or JQ1 (−). Cells were harvested at 48 h, EGFP and DsRed expression was quantified by flow cytometry. **a** The flow cytometry gating strategy used to identify  % EGFP and  % DsRed positive cells in a representative result from n = 3 independent experiments, each conducted in triplicate. **b** The mean percentage of spliced product DsRed/(DsRed + EGFP) from the 3 independent experiments is shown. Comparisons of each condition to JQ1 (+) were made using Friedman nonparametric test. Only statistically significant comparisons are shown *p < 0.05; **p < 0.01

In the absence of Tat, Bromodomain-containing protein 4 (BRD4), an acetylated histone binding protein with 2 bromodomains (BRD) that recognise acetylated lysine residues, plays an important role during transcriptional elongation by interacting and activating P-TEFb (positive transcription elongation factor b) [32, 33]. On the other hand, BRD4 can negatively impact HIV transcription via P-TEFb sequestration [34]. As JQ1 inhibits BRD4 activity by binding competitively to acetyl-lysine recognition motifs and releasing P-TEFb, we hypothesised that JQ1 might influence HIV transcription and splicing through BRD4. To further elucidate the interplay of JQ1 and BRD4 in regard to the transcriptional and splicing regulation of HIV RNA, we analysed the expression of HIV using the splicing reporter system after stimulation with 2 enantiomers of JQ1; S (+) and R (−) forms (Fig. 9).

As expected, treatment with JQ1 (−) analogue that does not interact with any bromodomain [35] abolished the JQ1 (+) effect on HIV transcription (Fig. 9a). Interestingly, as shown in Fig. 9b, a specific activation of HIV D4-A7 splicing with JQ1 (+), but not with the stereoisomer control JQ1 (−), was also observed in all cases (p = 0.0151; n = 3; Friedman nonparametric test) suggesting a role of BRD4 in mediating HIV D4-A7 RNA splicing via JQ1 (+).

## Discussion

A variety of LRAs are currently being investigated ex vivo and in vivo for their efficacy to induce virus production from latently infected rCD4+ T cells. However, not much is known about how these LRAs affect different aspects of the viral replication cycle, such as the behaviour of Tat protein in transcription and splicing. Given the ability of Tat to be post-translationally modified and the possibility that these modifications may differ under the influence of LRAs, we were interested to investigate how different classes of LRAs acted in combination with Tat.

In agreement with previous studies by Caputi’s team [8], our data show that wild-type (WT) Tat101 expression induced a change in HIV-1 splicing pattern, increasing the use of A7 3′ss and thus the expression of *rev* mRNAs. We also showed that the Tat K28 and K50/51 residues are essential for the additive effect between LRAs and Tat on transcription. Interestingly, K28 and K50 are highly conserved across HIV-1 and SIV [19]. These same Tat residues, in addition to R53, were also required for efficient RNA splicing. Additionally, in the presence of K28A, K50A, and K50/51A mutations that deny acetylation at these positions, JQ1 alone could rescue this defect in splicing. These data suggest that JQ1 can induce HIV RNA splicing independently of Tat, yet when Tat is present these residues are not essential. It is unclear if this positive activity of JQ1 and Tat extends to other alternatively spliced cellular RNAs.

Suboptimal levels of Tat protein or Tat function facilitate the maintenance of HIV latency where the addition of exogenous or co-transfected Tat can efficiently reactivate virus production from latent HIV [10, 36–40]. It remains unknown if Tat expression and appropriate post-translational modification can be induced by LRAs in rCD4+ T cells, particularly since the long non-coding RNA NRON, which is highly expressed in rCD4+ T cells, can target Tat for degradation [11]. Our results suggest that the Tat residues K28, K50 and K51, or the modifications that these basic amino acids acquire from cellular factors, are essential for Tat mediated HIV transcription and splicing.

**Fig. 10:**
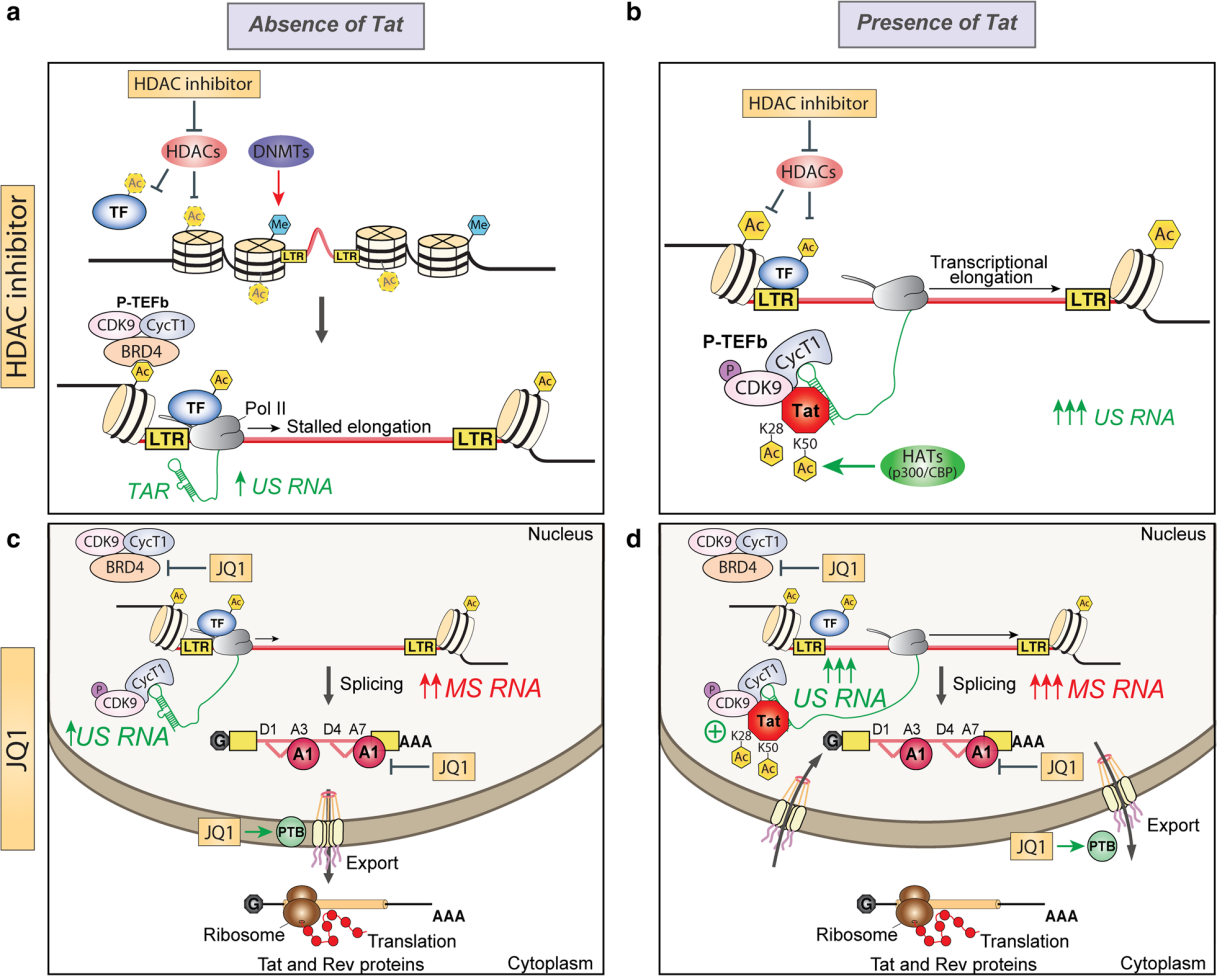
Proposed model of the effects of LRAs in combination with Tat on inducing HIV-1 transcription and splicing. **a** During latency, the nucleosomes surrounding the proviral 5′LTR are subject to repressive epigenetic modifications such as histone methylation and deacetylation induced by DNA methyltransferases (DNMT) and histone deacetylases (HDAC), respectively. Upon treatment with HDACi, histone acetylation by histone acetyltransferase (HAT) would induce chromatin decondensation and transcription factor (TF) mobilisation to the RNA polymerase II (Pol II). This subsequently relieves the transcriptional repression at the 5′LTR resulting in expression of low levels of HIV US RNA. **b** Tat expression can induce transcription elongation from quiescent LTR promoters by recruiting the positive transcription elongation factor b (P-TEFb), which comprises CDK9 and Cyclin T1 (CycT1), to the stalled RNA Pol II at the viral promoter. Acetylation of Tat lysine 28 (K28) by PCAF is required for high affinity binding of Tat to the TAR/SEC (Super elongation complex). In combination with Tat, HDACi such as vorinostat and panobinostat can reactivate latent proviruses by increasing the pool of active P-TEFb through CDK9 T-loop phosphorylation. **c** HIV expression may be further restricted by inefficient splicing and defects in nuclear export of multiply spliced HIV RNAs (MS RNA). Moreover, Bromodomain containing proteins such as BRD4 can compete with Tat for binding to P-TEFb. BRD inhibitor treatment (JQ1) may block the activity of BRD4, leading to release of P-TEFb and activation of HIV expression. Given our results, we suggest that JQ1 would further antagonise latency by enhancing HIV splicing through downregulation of hnRNP A1 levels, as well as promoting the export of MS RNAs to the cytoplasm by upregulating PTB expression. **d** Upon the initial rounds of Tat and Rev production following JQ1 treatment and P-TEFb release, Tat would enable transcriptional elongation through a strong positive feedback loop creating a pool of US HIV RNA that would be exported efficiently through Rev binding to the Rev-Responsive Element (RRE)

When WT Tat is expressed in the context of latent infection, we show that some LRAs more potently induce HIV transcription, than in the absence of Tat. This is due to the multifaceted behaviour of how Tat is controlled, in part, by the cellular environment and the ability of Tat to be post-translationally modified by a variety of cellular factors at different stages of the viral life cycle [14–27]. Although the effects of post-translational modifications on Tat are well characterised, it remains difficult to determine the exact trajectory of Tat in its feedback loop with regard to subcellular location, at what stage in viral replication and by what cellular factor/s that Tat is modified during the viral life cycle. Given these modifications can have positive or negative behaviour-changing effects on Tat function that can be transient or permanent, it makes sense that LRAs, which can act directly or indirectly on lysine or arginine residues, may enable the accumulation of a pool of modified Tat with altered behaviour that may change how Tat interacts and functions during latency reversal [14–27] (see schematic in Fig. 10).

HDACi can modestly activate transcription and their activity is enhanced in the presence of Tat, which could be due to the fact that Tat transactivation through the LTR is intricately controlled by lysine acetylation [14]. In particular, the acetylation of K28 is mediated by host histone acetyltransferases (HATs) p300, which strengthens the binding of Tat to the transactivation response (TAR) RNA element within the LTR to promote transcription [19, 21, 22]. Yet when K50 is acetylated by p300, this interaction enables the dissociation of the Tat•P-TEFb•TAR complex to transfer Tat onto the elongating RNA Pol II [19, 21]. Tat is hypothesized to control elongation rates by orchestrating the phosphorylation of the C-terminal domain (CTD) of RNA Pol II to enable the association and dissociation of transcribing RNAs and RNA-associated factors throughout elongation, as well as the control of the processivity and pausing of RNA Pol II to regulate viral splicing [8, 41, 42]. Given that the additive effect on transcription between HDACi and Tat is lost with the K28A and K50A mutants, we propose a model where HDACi act by promoting the acetylation of K28 and K50 to enhance transactivation by Tat-mediated assembly of the transcription complex (Fig. 10). However, acetylation of Tat at K28 may continuously promote the Tat:TAR interaction in a way that precludes splicing.

Using our in vitro model system to assess transcription and splicing, while JQ1 had a similar activity to HDACi in enhancing transcription, JQ1 also had a unique additional action in enabling splicing in the presence and absence of Tat101. JQ1 can act in both a Tat-dependent [43–46] and Tat-independent [47] manner to reverse latency, where the Tat-independent activity of JQ1 is crucial given that Tat protein is scarce in latency [10]. The positive effects of JQ1 on HIV splicing may be explained by its ability either to alter the level of *trans*-acting factors such as hnRNPs (heterogeneous nuclear ribonucleoproteins), which are involved in alternative splicing, mRNA stability, transcriptional and translational regulation [48], or to initiate the upregulation of the CDK9 subunit of P-TEFb [49] and AFF4 [44], as well as host genes crucial for chromatin reorganization and genes that influence posttranslational modifications of Tat [44]. We demonstrated that the lysine residues at K28, K50, and K50/51 were critical for splicing but JQ1 was able to rescue this defect. Our finding highlights the usefulness of compounds like JQ1 in situations where there are forms of Tat in the latent provirus that have attenuated activity, as defective proviruses rapidly accumulate during acute infection and shape the proviral landscape [50–52]. Therefore, bromodomain inhibitors may be more attractive compounds than HDACi for latency reversal.

JQ1 binds competitively to acetyl-lysine recognition domains as in BRD4 [34]. Besides its role in transcriptional elongation, the bromodomain protein BRD4 is involved in alternative splicing regulation as it interacts with JMJD6 (JmjC domain containing protein 6) that mediates 5′ hydroxylation of U2AF65, a major component of the spliceosomal complex important for 3′ splice site recognition [53, 54]. BRD4 can also regulate splicing following a heat shock response, as an increase in intron retention was observed upon BRD4 depletion [55]. Following JQ1 treatment, the expression of various host proteins is altered, including hnRNP A1 and PTB, which are known regulators of HIV-1 splicing and nuclear export of viral RNAs. HIV splice acceptor sites usage is strongly dependent on SR and hnRNP proteins [56–59]. Through cooperative binding to enhancer (ESE3) and silencer (ESS3a, ISS) elements nearby the viral A7 splice site, hnRNP A1, a known regulator of HIV-1 splicing, modulates *tat* and *rev* mRNA production [60]. Additionally, a lack of PTB (polypyrimidine tract-binding protein), also known as hnRNP I in resting CD4+ T cells has been implicated in nuclear retention of multiply spliced RNAs during latency [61]. An increase in the levels of PTB following JQ1 treatment would allow efficient export and expression of multiply spliced RNAs encoding for Tat and Rev. Given that changes in the balance of splicing can perturb viral replicative fitness and infectivity [29, 62], these alterations in splicing factors may have a major impact on the efficiency of virus production from latency. Whether through splicing and/or export, the ability of PTB to revert nuclear retention of multiply spliced RNAs in rCD4+ from patients on cART [61] indicates an mRNA processing restriction mechanism that is in place in rCD4+ T cells affecting Tat and Rev expression during latency. In fact, a new study revealed a series of blocks to HIV proximal elongation, distal transcription/polyadenylation (completion) and splicing (D4-A7) in CD4+ T cells from HIV infected patients on ART [63]. As viral Tat and Rev proteins play crucial roles in transcription initiation-elongation and nuclear export, multiply spliced transcripts (*tat* and *rev* mRNAs) may be of increased utility as a marker for viral rebound in patients after cART interruption [64] than unspliced RNA, which have been mainly used in clinical trials.

Recent study from Ott’s group has revealed that a short isoform of BRD4 promotes latency by engaging repressive SWI/SNF chromatin-remodelling complexes, which could be reversed by JQ1 treatment [65]. In synergy with protein kinase C (PKC) agonists such as Bryostatin-1, bromodomain and extra-terminal domain inhibitors (BETis) like JQ1, UMB-136 and OTX015 constitute highly effective LRA combinations capable of inducing robust increase in HIV mRNA expression, comparable to CD3/CD28 antibodies stimulation, in rCD4 T cells from infected individuals on ART without inducing global T cell activation. This reactivation occurs through binding of BETis with the long isoform of BRD4 and release of P-TEFb [46, 66–69]. In agreement with these previous reports, our data revealed JQ1 ability to induce BRD4 dependent HIV-1 transcription and D4-A7 RNA splicing.

Given the difficulty in measuring Tat protein and Tat activity ex vivo, there are several limitations in this study. We chose HEK293T cells to use the splicing reporter system given the feasibility of transfecting these cells with the multiple plasmids required to clearly visualize EGFP and DsRed expression, which proved more difficult in a T-cell line such as Jurkats. It would be interesting to test these Tat mutants and LRAs in the context of full-length virus in a primary cell model of latency or ex vivo given the different landscape of cellular factors in rCD4+ T cells compared to a cancer cell line that affect the capacity of a cell to reactivate a latent provirus. Differences in the availability of host transcription, elongation and splicing factors in a rCD4+ T cell may augment the results observed in this study. Primary resting cells lack sufficient levels of transcription and elongation factors that mainly remain in inhibitory complexes in the cytoplasm [70–72]. We predict our results may be more pronounced ex vivo given P-TEFb, which exists in very low levels in rCD4+ T cells [45, 73] can be released from its inhibitory complex with 7SK snRNP and HEXIM1 with the addition of an HDACi, JQ1 or Tat protein [45, 49, 72, 74, 75]. Finally, we did not specifically address whether these same effects on transcription and splicing are relevant in other cellular reservoirs such as long lived infected macrophages, or whether they can induce replication competent virus by measuring HIV RNA production in culture supernatant.

## Conclusions

A lack of Tat is important in maintaining latency in resting CD4+ T-cells and therefore Tat is not readily available during the initial reactivation of provirus. This will limit the potency of some LRAs, such as HDACi, which fail to induce splicing in the absence of Tat. In contrast, JQ1 which also acts in combination with Tat to activate transcription, can enable splicing even in the absence of Tat. In conclusion, the potency of an LRA to induce virus production is enhanced if Tat is present, as certain agents may work directly or indirectly through post-translational modifications of Tat. Strategies to increase Tat expression during latency reversal should be explored to fully activate the viral replication cycle and further enhance the potency of LRAs.

## Methods

### Tat101 mutants

Tat101^AD8^—Flag was inserted into pcDNA3.1 (−) vector (Invitrogen) cleaved by *Xba*I and *EcoR*I. Site-directed mutagenesis was used to insert alanine substitutions or conservative mutations at particular locations to remove Lysine or Arginine function within Tat (see Additional file 5: Table S1 for oligonucleotide sequences for each mutant). Mutagenesis PCR was performed using High-Fidelity PCR DNA Polymerase (Promega) according to the manufacturer’s instructions. The PCR reaction was performed as following: 98 °C 5 min, 30 cycles of 98 °C 30 s, 50 °C 30 s, 72 °C 7 min, and a final 72 °C 10 min. After treatment with *Dpn*I (NEB), amplified PCR product was purified with DNA gel extraction (Macherey–nagel nucleospin gel) and transformed into TOP10 competent *E. Coli* bacteria. Sequencing analysis confirmed the accuracy of cloning.

### Immunoblotting

HEK293T cells transfected with WT and mutants Tat plasmids, and then treated with JQ1 (1 μM) or DMSO diluent control, were lysed with RIPA buffer (50 mM Tris–HCl pH8, 150 mM NaCl, 1% IgePal, 1 mM EDTA) supplemented with protease inhibitor cocktail (Roche), followed by sonication and centrifugation at 12,000xg for 15 min at 4 °C. The amount of proteins in the cell lysate was determined by Bradford assay (BioRad). Equal amounts of each sample were loaded on 12.5% SDS-PAGE, transferred to a nitrocellulose membrane (0.45 μm BioRad) then blocked in 5% milk-PBS-T (0.1% Tween-20) for 1 h at room temperature. Blots were probed with anti-Flag (ab1162, abcam, 1/2500°), anti-GAPDH (#14C10, cell signalling, 1/1000°), anti-PTBP1 (clone 7, ThermoFischer Scientific, #325000, 1/500°) and anti-hnRNP A1 (clone 9H10, Santa Cruz, sc-56700, 1/25°). After several washes, the membrane was incubated with either 1/5000° goat anti-rabbit IgG (H + L) HRP (Invitrogen, Cat. No. 656120) or goat anti-mouse IgG (H + L) HRP (Invitrogen, Cat. No. 626520) for 1 h at RT. Blots were developed using Supersignal west pico chemiluminescent substrate (ThermoFisher Scientific) and visualized using the MF-ChemiBis 3.2 imaging system (DNR).

### Splicing reporter experiments

Splicing reporter experiments with pLTR.gp140/EGFP.Rev∆38/DsRed were performed as previously described [30, 31]. Briefly, 2 × 10^4^ HEK293T cells (human embryonic kidney cells that stably express the SV40 large T antigen; American Tissue Culture Collection) were seeded per well into 96-well plates with DMEM (Gibco) + 10% FBS with Penicillin (100U/ml)/Streptomycin (100 μg/ml) and cultured overnight. Cells were then transfected in the absence of antibiotics using Lipofectamine 2000 (ThermoFisher) with 400 ng of an LTR-driven splicing reporter pLTR.gp140/EGFP.Rev∆38/DsRed, 20 ng of pRev^NL4.3^, with or without wild type or mutant pTat101^AD8^ in triplicate wells per experiment on 5 separate occasions for n = 5. A matched empty tat vector pcDNA3.1 (−) was used for experiments without Tat. Cells were incubated for 5 h prior to treatment with DMSO (1:5000, #67-68-S Merck), vorinostat (0.5 μM, #10009929 Cayman Chemical or #S1047 Selleck Chemicals), panobinostat (30 nM, #P180500 TRC or #S1030 Selleck Chemicals), JQ1 (+) (1 μM, #11187 Cayman Chemical or #S7110 Selleck Chemicals), JQ1 (−) (1 μM, #11232 Cayman Chemical), chaetocin (30 nM, #C9492 Sigma), disulfiram (500 nM, #D3374 LKT or #S1680 Selleck Chemicals), or PMA/PHA (10 nM PMA, #16561-29-8 Sigma-Aldrich/10 μg/mL PHA, #HA15/R30852701 Remel). Cells were harvested at 48 h, stained with LIVE/DEAD Fixable Dead Cell Stain (Near-IR, Thermo Fisher Scientific) and then assessed for EGFP and DsRed expression by flow cytometry (LSRII, BD Biosciences). Optimal compensation was achieved using HEK293T cells expressing the individual fluorescent protein. A minimum of 10.000 viable cell events per sample was acquired. Data was analysed using *FlowJo version 10.0.8*. The gating strategy includes exclusion of debris and selection of single cells based on forward and side scatter. Size selected cells were subgated using the live/dead marker, followed by the identification of unspliced and spliced products as positive for EGFP and DsRed, respectively. For that, live cells were gated on EGFP and DsRed versus “dump channel” (violet). The CellTiter 96 Aqueous One Solution Cell Proliferation Assay (Promega) was also used following manufacturer’s instructions to determine the toxicity of the LRAs.

### Quantitative PCR of viral and human RNA species

Total RNA was extracted from cells using TRIzol (Invitrogen) following manufacturer instructions followed by RQ1 RNase-Free DNase (Promega, 2 U/μg) treatment for 30 min at 37 °C. One µg of DNase treated RNA was reverse-transcribed using Omniscript-reverse transcriptase (Qiagen), d(T)15 and random hexamer primers following the manufacturers specifications. HIV US, spliced and all viral RNA (copies/μl) were quantified by ddPCR. Briefly, the ddPCR reaction consisted of 12 μl 2 × ddPCR super mix for probes (no dUTP, Bio-Rad); 900 nM of each primer; 250 nM probe (FAM-MGBNFQ, Applied Biosystems, Additional file 5: Table S1) and 0.8-80 ng cDNA into a 24 μl final volume. Ribonuclease P/MRP 30 kDa (RPP30, dHsaCPE5038241), importin8 (IPO8, dHsaCPE5044719) and TATA-binding protein (TBP, dHsaCPE5058363) were used as reference genes (HEX, Bio-Rad) in multiplexed reactions with the HIV quantification. Following droplets generation (15,000-18,000 on average), thermal cycling was conducted as follows: 95 °C for 10 min, 40 cycles of 94 °C for 30 s and 60 °C for 60 s, followed by 98 °C for 10 min (ramp rate 2 °C/s for each step) on a C1000 Touch Thermal cycler (Bio-Rad). The droplets were subsequently read by a QX200 droplet-reader (Bio-Rad) and the data were analysed with *QuantaSoft 1.7.4 software*. A minus reverse transcriptase control (−RT) was included for each sample. The positive droplets were designated based on the −RT and the no template controls (NTC). Data of the triplicate wells per experiment were merged and the mean of the 4 independent assays was determined. The synthesized cDNA (10 ng) was also used as a template for semi-quantitative RT-PCR reactions to access exon inclusion and exclusion isoforms of *CD45*-*exon13*, *ATF2*-*exon6* and *ABI1*-*exon8,9* using primers listed in Additional file 5: Table S1. PCR products were resolved on a 2% agarose gel (TBE 1×) and visualized on a Syngene GBox imaging system.

### TZMbl experiments

TZMbl cells (NIH AIDS Reagent Program) were seeded into 96-well plates in DMEM (Gibco) + 10% FBS with Penicillin (100U/ml)/Streptomycin (100 μg/ml) and cultured overnight. Cells were then transfected in the absence of antibiotics using Lipofectamine 2000 (ThermoFisher) with or without WT or mutant pTat101^AD8^ (100 ng) in triplicates wells on 5 separate occasions for n = 5. Cells were harvested at 48 h and lysed with 35 μl of 1 × Passive Lysis Buffer (PLB, Promega), incubated for 5 min at RT before 5ul of each well was transferred to a CoStar 96-well white plate. The luciferase assay was performed as per the manufacturer’s protocol (Promega) by addition of 25 μl of LARII and quantified on a FLUOStar Omega microplate reader (BMG Labtech, Ortenburg, Germany).

### Statistical analyses

GraphPad PRISM version 7 software was used for statistical analyses. Paired *T* tests and 2-way ANOVA were used to compare values to DMSO and across all LRAs, as indicated.

## Additional files

**Additional file 1: Figure S1.** Cellular toxicity of LRAs. The CellTiter 96 Aqueous One Solution Cell Proliferation MTS assay was used to measure the toxicity of a panel of LRAs on HEK293T cells over a range of concentrations (31.25 to 1000 nM) for 48 h. VOR = vorinostat; PAN = panobinostat; CTN = chaetocin; DIS = disulfiram. The lines represent the mean + SD (n = 2)

**Additional file 2: Figure S2.** JQ1 increases EGFP and DsRed expression from an LTR-driven splicing reporter in the absence and presence of Tat. HEK293T cells were transfected with the pLTR.gp140/EGFP.Rev∆38/DsRed splicing reporter in the absence or presence of 100 ng of pTat101 (AD8)-Flag expression plasmid and then treated for 24 h with JQ1 (1 μM) or DMSO diluent control. Cells were harvested and portion analysed for either the percentage of cells expressing EGFP (unspliced, **A.**) or DsRed (spliced, **B.**) using flow cytometry, or HIV unspliced (US), spliced (D4-A7) and all viral RNA expression levels (copies/ul) by droplet digital PCR (ddPCR) (Fig. 3). The fold-change (FC) over DMSO of Live+ EGFP+ (**A.**), Live + DsRed + (**B.**) and percentage of spliced product DsRed/(DsRed + EGFP) (**C.**) were determined. Comparisons of each condition to DMSO were made using a paired *T* test. Only statistically significant comparisons are shown **p < 0.01; ***p < 0.001; ****p < 0.0001. The black lines represent the mean ± SEM (n = 4)

**Additional file 3: Figure S3.** Cellular and HIV RNA levels following JQ1 treatment. **A.** Absolute quantification of *RPP30*, *IPO8* and *TBP* cellular mRNAs (copies/μl) were performed using total RNAs derived from transfected HEK293T cells with the pLTR.gp140/EGFP.Rev∆38/DsRed splicing reporter in the absence and presence of 100 ng of pTat101 (AD8)-Flag expression plasmid and treated with JQ1 (1 μM) or DMSO diluent control. **B.** HIV unspliced (US), spliced (D4-A7) and all viral RNA expression levels (copies/ul) were quantified by droplet digital PCR (ddPCR) and normalized over the 3 reference genes. Comparisons of each condition to DMSO were made using a paired *T* test. Only statistically significant comparisons are shown *p < 0.05; **p < 0.01; ***p < 0.001; ****p < 0.0001. The black lines represent the mean ± SEM (n = 4)

**Additional file 4: Figure S4.** Some Tat mutants reduce the additive effect with LRAs on transcription. HEK293T cells were transfected with the pLTR.gp140/EGFP.Rev∆38/DsRed splicing reporter and 100 ng of pTat101 (AD8)-Flag with specific mutations; K28A (**A.**), K50A (**B.**), K50/51A (**C.**), K71A (**D.**), R53A (**E.**) and were treated with a panel of LRAs. Cells were harvested at 48 h and EGFP expression from the US mRNA was quantified using flow cytometry and represented as  % EGFP positive cells. Comparisons of each condition to DMSO were made using 2-way ANOVA test. Only statistically significant comparisons are shown * p < 0.05; ** p < 0.01; *** p < 0.001; **** p < 0.0001. The black lines represent the mean ± SEM (n = 5). DMSO (1:5000), VOR = vorinostat (0.5 μM), PAN = panobinostat (30 nM), JQ1 (+) (1 μM), CTN = chaetocin (30 nM), DIS = disulfiram (500 nM), or PMA/PHA = phorbol myristate acetate/phytohaemagglutinin (10 nM PMA, 10 μg/mL PHA)

**Additional file 5: Table S1.** Oligonucleotides used in this study for
