# Supplementary figures and images for: HIV latency reversing agents act through Tat post translational modifications
Source: Retrovirology. 2018 May 11;15:36. doi: 10.1186/s12977-018-0421-6 (PMC5948896; doi:10.1186/s12977-018-0421-6)

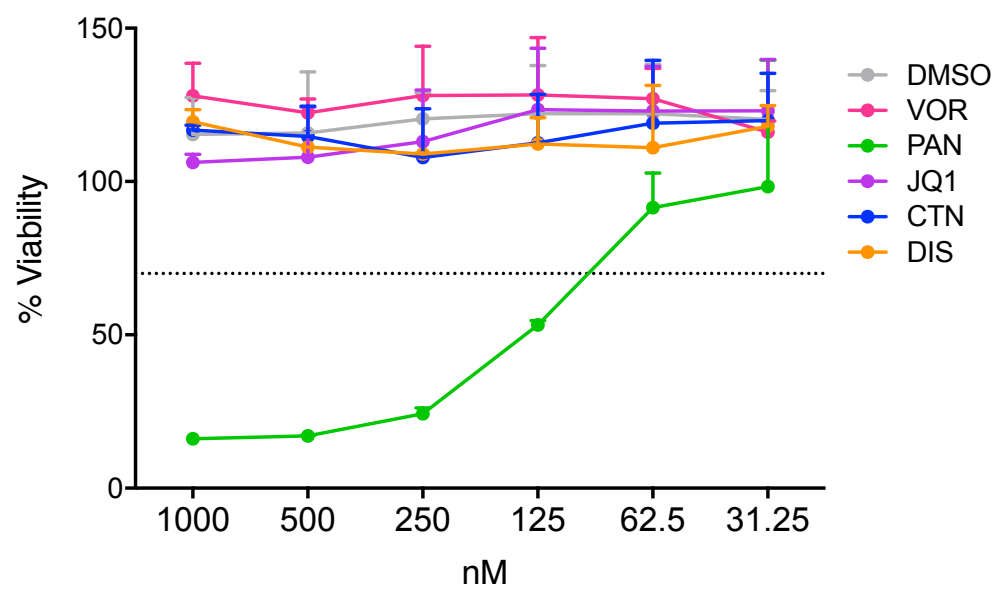

**Figure S1**

Supplement: Supplementary file 1 — Additional file 1: Figure S1. Cellular toxicity of LRAs. The CellTiter 96 Aqueous One Solution Cell Proliferation MTS assay was used to measure the toxicity of a panel of LRAs on HEK293T cells over a range of concentrations (31.25 to 1000 nM) for 48 h. VOR = vorinostat; PAN = panobinostat; CTN = chaetocin; DIS = disulfiram. The lines represent the mean + SD (n = 2) [file 12977_2018_421_MOESM1_ESM.pdf]

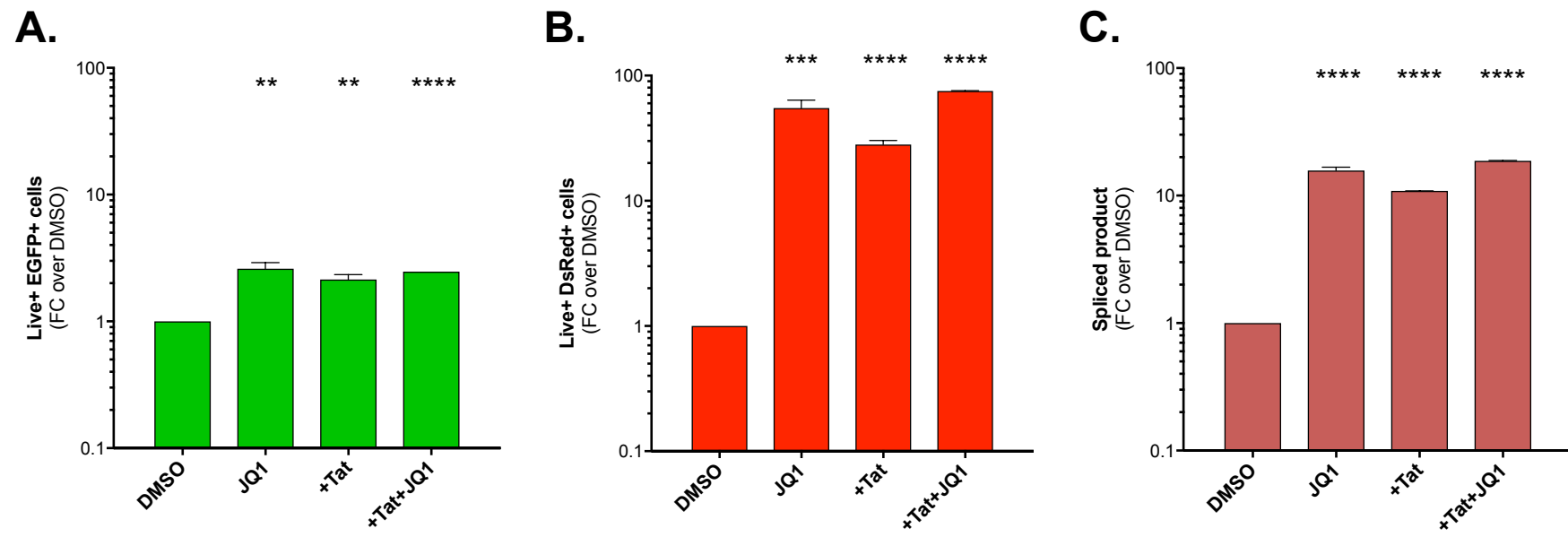

**Figure S2**

Supplement: Supplementary file 2 — Additional file 2: Figure S2. JQ1 increases EGFP and DsRed expression from an LTR-driven splicing reporter in the absence and presence of Tat. HEK293T cells were transfected with the pLTR.gp140/EGFP.Rev∆38/DsRed splicing reporter in the absence or presence of 100 ng of pTat101 (AD8)-Flag expression plasmid and then treated for 24 h with JQ1 (1 μM) or DMSO diluent control. Cells were harvested and portion analysed for either the percentage of cells expressing EGFP (unspliced, A.) or DsRed (spliced, B.) using flow cytometry, or HIV unspliced (US), spliced (D4-A7) and all viral RNA expression levels (copies/ul) by droplet digital PCR (ddPCR) (Fig. 3). The fold-change (FC) over DMSO of Live+ EGFP+ (A.), Live + DsRed + (B.) and percentage of spliced product DsRed/(DsRed + EGFP) (C.) were determined. Comparisons of each condition to DMSO were made using a paired T test. Only statistically significant comparisons are shown **p < 0.01; ***p < 0.001; ****p < 0.0001. The black lines represent the mean ± SEM (n = 4) [file 12977_2018_421_MOESM2_ESM.pdf]

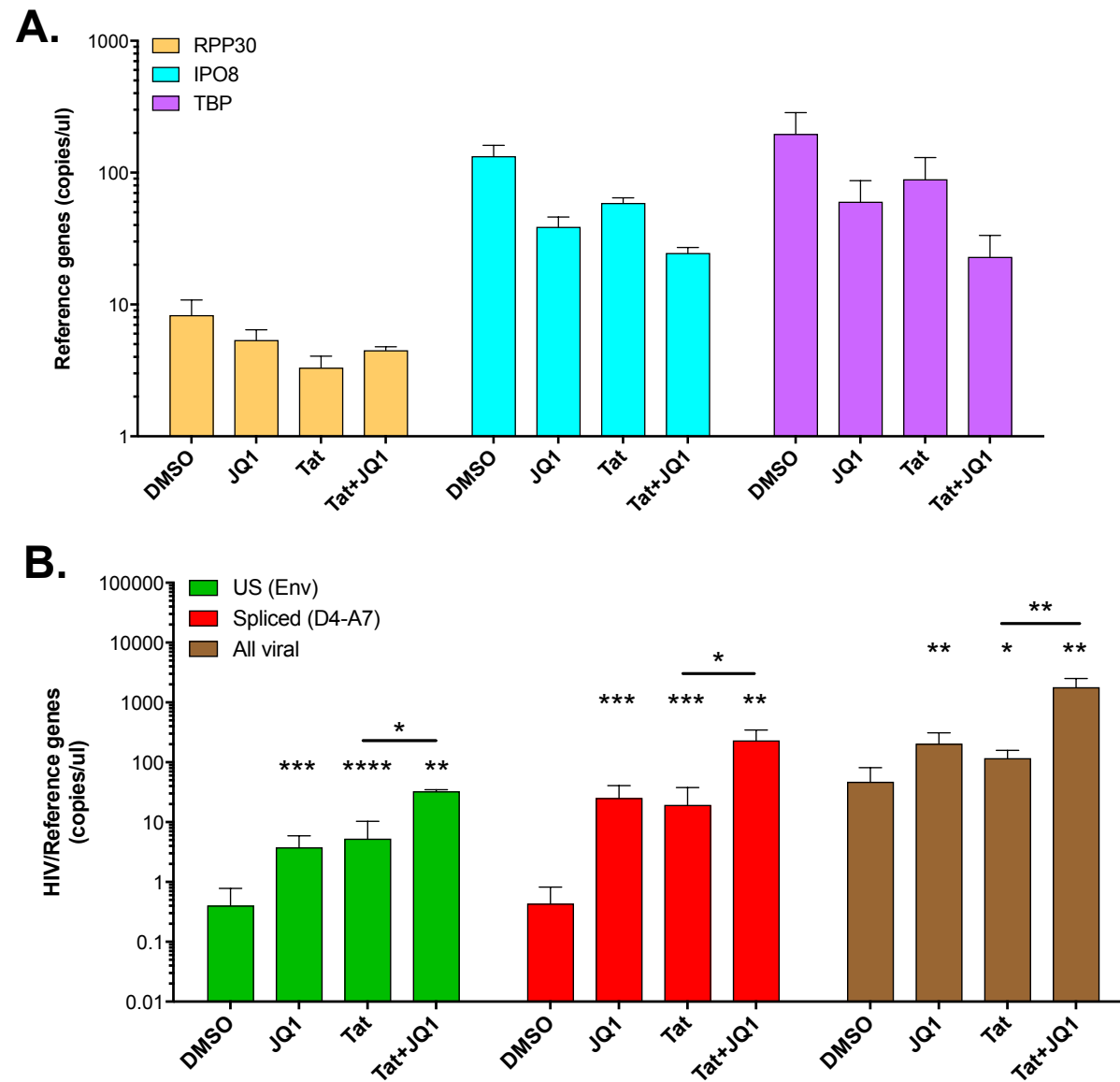

**Figure S3**

Supplement: Supplementary file 3 — Additional file 3: Figure S3. Cellular and HIV RNA levels following JQ1 treatment. A. Absolute quantification of RPP30, IPO8 and TBP cellular mRNAs (copies/μl) were performed using total RNAs derived from transfected HEK293T cells with the pLTR.gp140/EGFP.Rev∆38/DsRed splicing reporter in the absence and presence of 100 ng of pTat101 (AD8)-Flag expression plasmid and treated with JQ1 (1 μM) or DMSO diluent control. B. HIV unspliced (US), spliced (D4-A7) and all viral RNA expression levels (copies/ul) were quantified by droplet digital PCR (ddPCR) and normalized over the 3 reference genes. Comparisons of each condition to DMSO were made using a paired T test. Only statistically significant comparisons are shown *p < 0.05; **p < 0.01; ***p < 0.001; ****p < 0.0001. The black lines represent the mean ± SEM (n = 4) [file 12977_2018_421_MOESM3_ESM.pdf]

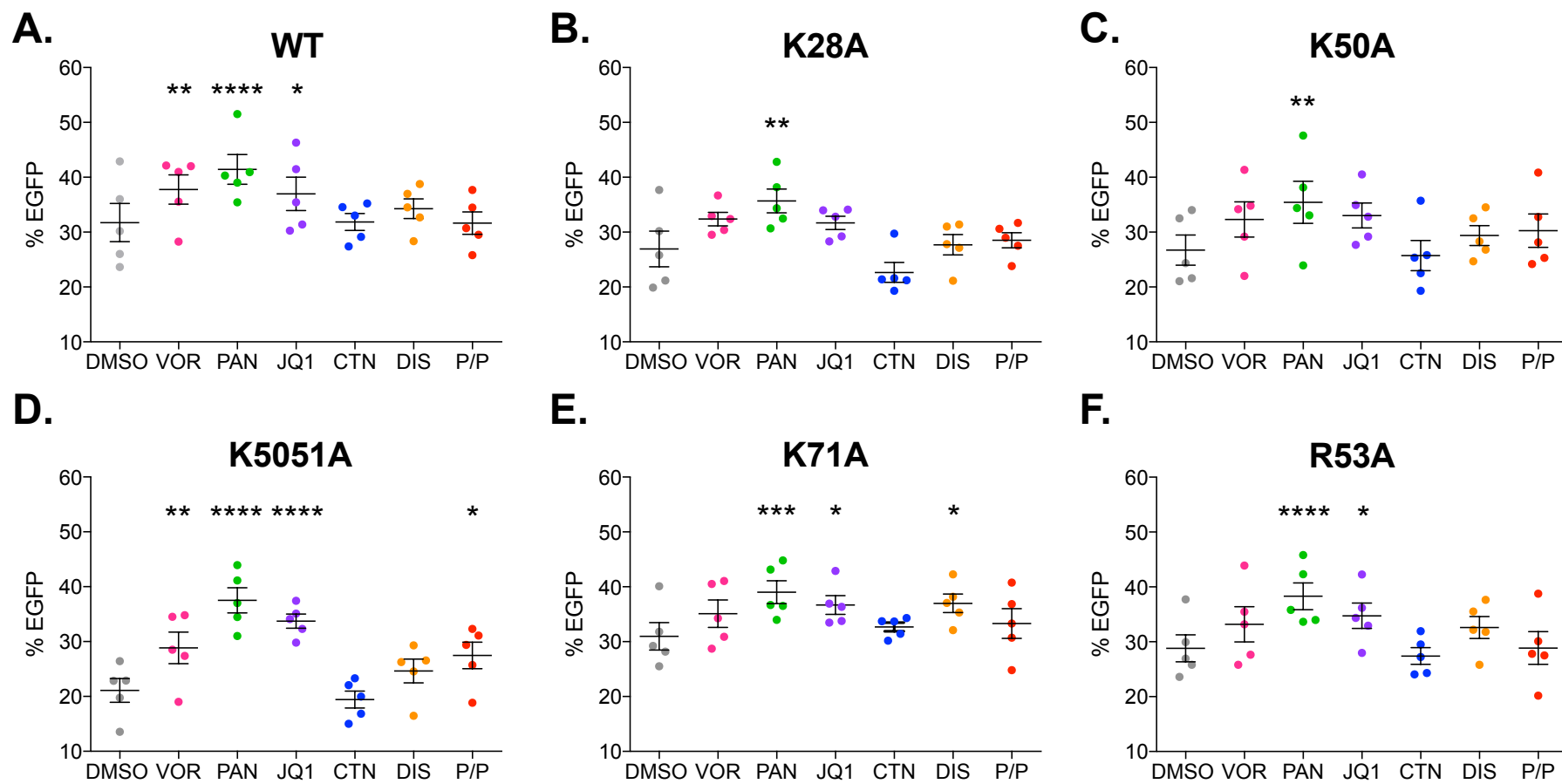

**Figure S4**

Supplement: Supplementary file 4 — Additional file 4: Figure S4. Some Tat mutants reduce the additive effect with LRAs on transcription. HEK293T cells were transfected with the pLTR.gp140/EGFP.Rev∆38/DsRed splicing reporter and 100 ng of pTat101 (AD8)-Flag with specific mutations; K28A (A.), K50A (B.), K50/51A (C.), K71A (D.), R53A (E.) and were treated with a panel of LRAs. Cells were harvested at 48 h and EGFP expression from the US mRNA was quantified using flow cytometry and represented as % EGFP positive cells. Comparisons of each condition to DMSO were made using 2-way ANOVA test. Only statistically significant comparisons are shown * p < 0.05; ** p < 0.01; *** p < 0.001; **** p < 0.0001. The black lines represent the mean ± SEM (n = 5). DMSO (1:5000), VOR = vorinostat (0.5 μM), PAN = panobinostat (30 nM), JQ1 (+) (1 μM), CTN = chaetocin (30 nM), DIS = disulfiram (500 nM), or PMA/PHA = phorbol myristate acetate/phytohaemagglutinin (10 nM PMA, 10 μg/mL PHA) [file 12977_2018_421_MOESM4_ESM.pdf]
