# Supplementary material for: HIV latency reversing agents act through Tat post translational modifications
Source: Retrovirology. 2018 May 11;15:36. doi: 10.1186/s12977-018-0421-6 (PMC5948896; doi:10.1186/s12977-018-0421-6)
Supplement: Supplementary file 5 — Additional file 5: Table S1. Oligonucleotides used in this study for [file 12977_2018_421_MOESM5_ESM.docx]

**Table S1**. Oligonucleotides used in this study for

| **Oligos** | **Sequence (5' -> 3')** | **Use** |
| --- | --- | --- |
| **Cloning and site-directed mutagenesis of Tat101(AD8)-Flag** | | |
| **odp3265** | GCTTGTAACAATTGCTATTGTGCAAAGTGTTGCTTTCATTGCCAAGTTT | 5' Tat K28A (AAA->GCA) in pcDNA3.1::Tat101^AD8^ |
| **odp3266** | AAACTTGGCAATGAAAGCAACACTTTGCACAATAGCAATTGTTACAAGC | 3' Tat K28A (AAA->GCA) in pcDNA3.1::Tat101^AD8^ |
| **odp3267** | GGCTTAGGCATCTCCTATGGCAGGGCGAAGCGGAGACAGCGACGAAGAGCTC | 5' Tat K50A (AAG->GCG) in pcDNA3.1::Tat101^AD8^ |
| **odp3268** | GAGCTCTTCGTCGCTGTCTCCGCTTCGCCCTGCCATAGGAGATGCCTAAGCC | 3' Tat K50A (AAG->GCG) in pcDNA3.1::Tat101^AD8^ |
| **odp3269** | GGCTTAGGCATCTCCTATGGCAGGGCGGCGCGGAGACAGCGACGAAGAGCTC | 5' Tat K50/51A (AAGAAG->GCGGCG) in pcDNA3.1::Tat101^AD8^ |
| **odp3270** | GAGCTCTTCGTCGCTGTCTCCGCGCCGCCCTGCCATAGGAGATGCCTAAGCC | 3' Tat K50/51A (AAGAAG->GCGGCG) in pcDNA3.1::Tat101^AD8^ |
| **odp 3427** | TCAGACTCATCAAGTTTCTCTATCAGCGCAACCCACCTCCCAGCCCCGAGGGG | 5' Tat K71A (AAG->GCG) in pcDNA3.1::Tat101^AD8^ |
| **odp 3428** | CCCCTCGGGGCTGGGAGGTGGGTTGCGCTGATAGAGAAACTTGATGAGTCTGA | 3' Tat K71A (AAG->GCG) in pcDNA3.1::Tat101^AD8^ |
| **odp3403** | ATGGCAGGAAGAAGCGGGCACAGCGACGAAGAGCTCC | 5' Tat R53A (AGA->GCA) in pcDNA3.1::Tat101^AD8^ |
| **odp3404** | GGAGCTCTTCGTCGCTGTGCCCGCTTCTTCCTGCCAT | 3' Tat R53A (AGA->GCA) in pcDNA3.1::Tat101^AD8^ |
| **odp3405** | ATAAATCTAGA**ATG**GAGCCAGTAGATCCTAGACTAG | 5' Tat101^AD8^ into XbaI-EcoRI of pcDNA3.1(-) with addition of a **Flag tag = DYKDDDDK**. |
| **odp3406** | ATAAAGAATTC**CTATTTATCATCATCATCTTTATAATC**ATCGCACGGATCTGTCTCTG | 3' Tat101^AD8^ into XbaI-EcoRI of pcDNA3.1(-) with addition of a **Flag tag = DYKDDDDK**. |
| **Absolute quantification by ddPCR** | | |
| **odp3167** | ACTTGCTCAATGCCACAGCCA | Oligo 5' to amplify all viral mRNAs (150 bp product) by ddPCR |
| **odp3168** | ACAGGGCTTGGAAAGGATTT | Probe to amplify all viral mRNAs (150 bp product) by ddPCR. 6-FAM/MGBNFQ |
| **odp3169** | ACAGGGCTTGGAAAGGATTT | Oligo 3' to amplify all viral mRNAs (150 bp product) by ddPCR. |
| **odp3113** | CAGAACAGTCAGACTCATCAA | Oligo 5' to amplify unspliced and spliced RNAs by ddPCR. |
| **odp3168** | TCTATCAAAGCAACCCACCTCCCAATC | Probe to amplify spliced RNA by ddPCR (113 bp product); binds across the D4-A7 junction. 6-FAM/MGBNFQ. |
| **odp3169** | TGAAGCGCATGAACTCCTT | Oligo 3' to amplify spliced RNA (Drev38-dsRed) by ddPCR (113 bp product). |
| **odp3117** | CACTTGTGGAGATGGGGGTGGAAAT | Probe to amplify unspliced RNA by ddPCR (285 bp product); binds downstream D4 5’ss. 6-FAM/MGBNFQ. |
| **odp3118** | ATCCCAAGGAGCATGGTG | Oligo 3' to amplify unspliced RNA by ddPCR (285 bp product). |
| **Semi-quantitative RT-PCR** | | |
| **odp3691** | GCAGTAATTTGTGTTGTCCCG | 5’ CD46, inclusion of exon 13 200bp; skipping 107bp. |
| **odp3692** | GGAGTGGTTGATTTAGTCTGG | 3’ CD46, inclusion of exon 13 200bp; skipping 107bp. |
| **odp3701** | TTCTATGTACTGCGCCTGGA | 5’ ATF2, inclusion of exon 6 180bp; skipping 61bp. |
| **odp3702** | GGTGTTGCAAGAGGGGATAA | 3’ ATF2, inclusion of exon 6 180bp; skipping 61bp. |
| **odp3709** | TGGAGGAAGTGGAAGTCGAG | 5’ ABI1, double inclusion 424bp; double exclusion 166bp; inclusion exon9 343bp; inclusion exon8 247bp. |
| **odp3710** | GGGAGGTGGAGAGTCATCAA | 3’ ABI1, double inclusion 424bp; double exclusion 166bp; inclusion exon9 343bp; inclusion exon8 247bp. |
